# *Veni, vidi, vici*: the success of *wtf* meiotic drivers in fission yeast

**DOI:** 10.1002/yea.3305

**Published:** 2018-02-21

**Authors:** José Fabricio López Hernández, Sarah E. Zanders

**Affiliations:** ^1^ Stowers Institute for Medical Research Kansas City MO 64110 USA; ^2^ Department of Molecular and Integrative Physiology University of Kansas Medical Center Kansas City KS 66160 USA

**Keywords:** infertility, meiosis, meitoic drive, Schizosaccharomyces, speciation

## Abstract

Meiotic drivers are selfish DNA loci that can bias their own transmission into gametes. Owing to their transmission advantages, meiotic drivers can spread in populations even if the drivers or linked variants decrease organismal fitness. Meiotic drive was first formally described in the 1950s and is thought to be a powerful force shaping eukaryotic genomes. Classic genetic analyses have detected the action of meiotic drivers in plants, filamentous fungi, insects and vertebrates. Several of these drive systems have limited experimental tractability and relatively little is known about the molecular mechanisms of meiotic drive. Recently, however, meiotic drivers were discovered in a yeast species. The Schizosaccharomyces pombe wtf gene family contains several active meiotic drive genes. This review summarizes what is known about the wtf family and highlights its potential as a highly tractable experimental model for molecular and evolutionary characterization of meiotic drive.

## 1 INTRODUCTION

Allele transmission through meiosis is generally thought to be fair. One of the first things nascent geneticists are taught is that Aa heterozygotes pass both ‘big A’ and ‘little a’ to half of their offspring. This rule of heredity was first recognized by the monk Gregor Mendel and is generally thought to be so rigidly followed that it is commonly known as Mendel's *law* of segregation (Abbott & Fairbanks, 2016). There is, however, tremendous evolutionary incentive for alleles to act selfishly and break this law. If ‘little a’ forces its own transmission to more than half of the gametes, it could spread to fixation in the population. This selfish behaviour is known as meiotic drive and is widespread in eukaryotes including plants, fungi, insects and mammals (Lindholm et al., 2016).

The term meiotic drive was coined 60 years ago to specifically describe biased segregation into the one gamete made during asymmetric (female) meiosis (Sandler & Novitski, 1957). Maize chromosomal knobs, for example, bias chromosome segregation to be preferentially transmitted into the female gamete whereas their competing alleles are lost in the polar bodies (Rhoades, 1942). This type of meiotic drive has also been observed in monkeyflowers, mice and humans (Didion et al., 2015; Fishman & Saunders, 2008; Ottolini et al., 2015; Pardo‐Manuel de Villena & Sapienza, 2001).

The term meiotic drive is also widely used to describe the actions of other selfish alleles that act to bias their own transmission into gametes without directly affecting chromosome segregation in meiosis (Lindholm et al., 2016; Zimmering, Sandler, & Nicoletti, 1970). These drivers act by causing the death or malfunction of gametes that fail to inherit them and can thus be called ‘gamete‐killers’ or ‘killers.’ The best understood of these is arguably the *het‐s* allele of *Podospora anserina* that encodes a prion protein and drives against the *het‐S* allele. HET‐s prions induce a conformational change in HET‐S proteins expressed in the spores that inherit the *het‐S* locus. Those altered HET‐S proteins then form a pore that disrupts the plasma membrane, causing cell death (Dalstra, Swart, Debets, Saupe, & Hoekstra, 2003; Seuring et al., 2012). Not all gamete‐killers, however, work the same way. The t‐haplotype driver in mouse interferes with the motility of the sperm that do not inherit the selfish locus by disrupting a Rho GTPase signalling cascade (Bauer, Willert, Koschorz, & Herrmann, 2005; Schimenti, 2000). Killer meiotic drivers have been observed in a wide range of eukaryotes including plants, insects, mice and filamentous fungi (Burt & Trivers, 2006; Larracuente & Presgraves, 2012; Lindholm et al., 2016; Turner & Perkins, 1979; Yang et al., 2012). Until recently, however, meiotic drivers were conspicuously absent in yeasts.

Meiotic drivers can be costly to the organisms that carry them. Selfish alleles can directly contribute to infertility by destroying gametes. Drivers can also promote the maintenance and spread of linked mal‐adapted (e.g. disease causing) alleles in a population. In fact, drive alleles are often linked to recessive mutations that cause infertility or non‐viability (Dyer, Charlesworth, & Jaenike, 2007; Larracuente & Presgraves, 2012; Schimenti, 2000). Owing to these fitness costs, unlinked suppressors that prevent meiotic drive should be favoured by selection (Burt & Trivers, 2006; Crow, 1991). This generates a genetic conflict between drivers and suppressors in which both sides are predicted to rapidly evolve (McLaughlin Jr & Malik, 2017). As drivers exploit gametogenesis, it is likely that suppressors will be co‐opted from amongst gametogenesis genes. This could force the genome to make costly tradeoffs in which variants that are suboptimal for their role in gametogenesis are selected owing to their ability to suppress drive. Understanding the molecular tactics used by meiotic drivers to gain a transmission bias will probably provide critical insights into the processes of gametogenesis and the causes of infertility. In addition, analysing the molecular evolutionary arms races fostered by meiotic drivers will augment understanding of the forces shaping genome evolution.

Although many drive systems have been identified, the actual driving alleles underlying many meiotic drive systems are unknown. Even in most cases where some or all the genes required for drive are known, the molecular mechanisms the genes use to enact drive remain uncharacterized. One factor that has historically limited progress in the field is the genetic complexity of many identified drive systems. Many drive systems require multiple genes and the genes are often associated with chromosome inversions (Bauer et al., 2005; Dyer et al., 2007; Harvey et al., 2014). These inversions prevent drive loci from being disrupted by recombination, but they also hinder efforts to map key genes. In addition to the complexity of drive loci, many drive systems have been identified in organisms with historically limited genetic tools (Dyer et al., 2007; Phadnis & Orr, 2009; Presgraves, Severance, & Wilkinson, 1997). However, the recent discovery of meiotic drive in fission yeast, which provides a nearly unparalleled level of experimental tractability, should greatly facilitate addressing questions of how meiotic drive genes work and drive genome evolution (Hu et al., 2017; Nuckolls et al., 2017; Zanders et al., 2014).

## 2 DISCOVERY OF MEIOTIC DRIVE IN FISSION YEAST

Most genetic experiments are carried out in isogenic or inbred organisms. This is especially true in the fission yeast *Schizosaccharomyces pombe* in which almost all commonly used laboratory stocks derive from a single strain isolated in France from grape juice in 1921 (Hu, Suo, & Du, 2015). Urs Leupold then took this isolate and developed it into a genetic system (Fantes & Hoffman, 2016). This isogeny has allowed generations of *pombe* geneticists to control for background effects and focus on the phenotypes caused by a given variant. The phenotypes of meiotic drivers, however, are invisible in isogenic organisms as they require heterozygosity to exhibit drive. Therefore, it is not surprising that meiotic drivers in fission yeast went undetected for decades.

Recent work exploring the genetic and phenotypic diversity of additional *pombe* isolates led to the discovery of yeast meiotic drivers. Not surprisingly, Amar Klar was a pioneer in this area. Klar's group identified a fission yeast variant in fermented tea and classified it as a distinct biological species, *S. kambucha*, because they found that laboratory *S. pombe/S. kambucha* hybrids are sterile (Singh & Klar, 2002). *S. kambucha* was subsequently sequenced and found to be highly similar, ~99.5% average DNA sequence identity genome‐wide, to the common laboratory isolate of *S. pombe* (Rhind et al., 2011).

The rapid evolution of reproductive isolation between isolates of *S. pombe* suggested the existence of genetic conflict during gametogenesis. This hypothesis was supported by work in Harmit Malik's laboratory that demonstrated the existence of spore‐killing meiotic drive loci with varying strengths in *S. kambucha*, at least one on each of the three chromosomes. This work also posited the existence of a meiotic drive locus on *S. pombe* chromosome 3 (Zanders et al., 2014).

The identity of the first cloned yeast meiotic drive genes was reported in back‐to‐back papers this year. Both groups used next‐generation sequencing‐assisted recombination mapping approaches to identify the drive loci. Nuckolls et al. (2017) returned to the *S. pombe*/*S. kambucha* hybrids and mapped two meiotic drive loci on *S. kambucha* chromosome 3. This work revealed a complex landscape of drivers in both strains. They also found evidence consistent with at least one drive suppressor – one region of *S. pombe* exhibited drive only when isolated in an otherwise *S. kambucha* background (i.e. in the absence of the putative suppressor).

Hu et al. (2017) took an analogous approach by mapping the cause of infertility in hybrids generated by mating the laboratory *S. pombe* strain to CBS5557, another yeast isolate from Spain. Like *S. kambucha*, CBS5557 is nearly identical (~99.5% genome average DNA sequence identity) to the laboratory *S. pombe* isolate. Hu et al. found that gamete killing meiotic drive also contributed to infertility in the laboratory *S. pombe*/CBS5557 hybrids. They also identified two meiotic drive loci on chromosome 3 of CBS5557 (Hu et al., 2017).

Both groups identified distinct meiotic drive genes that are members of the previously uncharacterized *wtf* gene family. The *S. kambucha* genes were named *wtf4* and *wtf28*, whereas the CBS5557 genes were called *cw9* and *cw27* (Hu et al., 2017; Nuckolls et al., 2017). The *wtf* gene family owes its catchy name to the family's association with long terminal repeats (LTRs) of the Tf transposons (with Tf transposon). Hu et al. (2017) demonstrated that this LTR association is not functionally important by showing that both *cw9* and *cw27* caused drive when their flanking LTRs were deleted.

## 3 MOLECULAR MECHANISMS OF *WTF* DRIVERS

The *wtf* meiotic drive genes act by causing the death of spores that fail to inherit them from a heterozygote. Spore death and allele transmission bias are not observed in homozygotes (*wtf+* or *wtf−*). All four described genes can cause drive when introduced to an ectopic locus in the laboratory *S. pombe*, indicating that they are each self‐sufficient for executing drive (Hu et al., 2017; Nuckolls et al., 2017). This is similar to other described single‐gene drive systems, *het‐s* and the *Spok* genes, of the *Podospora anserina* fungus (Dalstra et al., 2003; Grognet, Lalucque, Malagnac, & Silar, 2014). The self‐sufficiency of these drivers is remarkable in that a single gene can both distinguish self from non‐self, and destroy non‐self spores.

**Figure 1:**
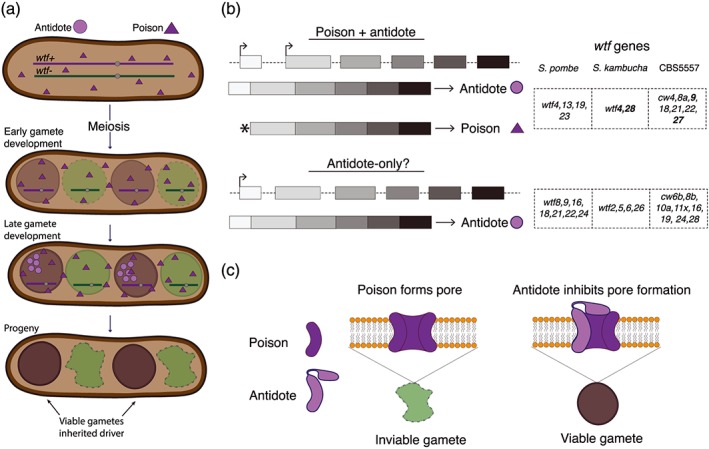
Meiotic drive in fission yeast. (a) The driving *Sk wtf4* gene makes two proteins: a *trans*‐acting poison that is first expressed prior to the meiotic divisions and a gamete‐specific antidote expressed after gamete (spore) individualization. The gametes that do not carry the *wtf* driver allele are destroyed. (b) The *Sk wtf4* poison and antidote proteins are made using alternative transcripts. Other *wtf* genes appear to share the ability to make two transcripts (top). Verified drive genes are shown in bold. A second class of *wtf* genes (bottom) appears to encode only a long transcript that is similar to the antidote transcript of *Sk wtf4*. (c) Model describing the hypothesized mechanisms of how the Wtf^poison^ protein kills and how the Wtf^antidote^ proteins neutralize the poisons [Colour figure can be viewed at http://wileyonlinelibrary.com]

Nuckolls et al. (2017) explored how *wtf* genes could accomplish these tasks using *S. kambucha wtf4* as a model (Figure 1). To elucidate the mechanism of *wtf4*, they created separation‐of‐function alleles. This work revealed that *wtf4* encodes two distinct proteins, a poison and an antidote. In a previously undescribed drive mechanism, these two proteins are made using alternative transcriptional and translational start sites. The Wtf4^poison^ protein is encoded in a transcript that includes exons 2–6, plus two amino acids upstream of exon 2. This protein is first expressed prior to the meiotic divisions and all four spores generated by a heterozygote (*wtf4+/wtf4−*) are poisoned. The poison is highly effective and most spores exposed to the poison in the absence of an antidote die. The spores that inherit *wtf4+*, however, also express the Wtf4^antidote^ from a longer message that includes exons 1–6. The Wtf4^antidote^ protein is expressed only after spore individualization and the protein largely remains within the cells that encode the *wtf4+* locus. This coordinated expression of poison and antidote proteins results in the targeted destruction of *wtf4−* spores (Figure 1; Nuckolls et al., 2017).

It is likely that the other identified driving *wtf* genes also act via a similar mechanism. Two transcripts are common amongst *wtf* genes and the other three *bona fide wtf* drive genes also contain a potential alternative translational start site near the beginning of exon 2 (Hu et al., 2017; Kuang, Boeke, & Canzar, 2016; Nuckolls et al., 2017). In addition, Hu et al. (2017) found that deletions of the regions upstream of exon 1 of *cw9* and *cw27* generated poison‐only separation of function alleles. These results are consistent with the deletions disrupting expression of the antidote proteins from exons 1–6, but not affecting the expression of the poison proteins from exons 2–6.

It is currently unknown how the Wtf poison proteins kill spores or how the Wtf antidote proteins neutralize the poisons. It seems likely that a critical dose of the poison is required for toxicity as low levels of the poison are detectable well before the appearance of the antidote protein (Nuckolls et al., 2017). In addition, the four spores produced by *cw27+/cw27−* heterozygotes initially all look the same by electron microscopy. Later in spores maturation, the *cw27−* spores become markedly different (Hu et al., 2017). Similarly, in *wtf4*+/*wtf4*− asci, the doomed spores are misshapen and the membranes become permeable to the dye propidium iodide (Nuckolls et al., 2017).

The sequences of the poison and antidote proteins offer limited clues about their mechanisms. The proteins contain multiple predicted transmembrane domains. It is possible that Wtf poisons kill cells by oligomerizing to form a pore in a vital membrane during spore development, analogous to bacterial protein toxins (Hu et al., 2017; Lee & Lee, 2016; Nuckolls et al., 2017). Owing to the shared sequences between the poison and antidote proteins, the Wtf antidotes could join the poison oligomers and disrupt pore formation or potentially actively promote destruction of the poison proteins (Figure 1c). If the ability of antidotes to suppress poisons does rely on shared amino acid sequences, mutations that generate novel poisons would simultaneously generate compatible antidotes, allowing fast co‐evolution in the overlapped sequences. This mechanism could have facilitated the expansion and diversification of the *wtf* gene family.

## 4 EVOLUTION *WTF* DRIVERS

The theoretical literature examining meiotic driver evolution is extensive, but to our knowledge none of these analyses predicted a family of genes as successful as the *wtf* genes (Burt & Trivers, 2006). The origins of the *wtf* gene family are unknown, but the family probably arose recently within fission yeasts. The gene family exhibits rapid evolution with dynamic gene copy numbers and DNA sequence changes between syntenic loci in different isolates (Hu et al., 2017; Nuckolls et al., 2017). The number of *wtf* genes varies between isolates with 25 *wtf* genes in the laboratory isolate of *S. pombe* at 20 different locations and 32 *wtf* genes in the assembled CBS5557 genome at 23 locations, including 3 locations not found in the laboratory isolate (Bowen, Jordan, Epstein, Wood, & Levin, 2003; Hu et al., 2017). The genes are found as singletons, pairs or triplets at each location. In both isolates, the genes are grossly enriched on chromosome 3. Twenty‐three of the *wtf* genes are on chromosome 3 in the laboratory isolate. This is remarkable because chromosome 3 is the smallest of the three chromosomes.

Each studied strain contains multiple genes that appear capable of encoding a driver with two proteins: an antidote (exons 1–6) and a poison (exons 2–6). The *S. pombe* reference genome, for example, contains four such genes: *wtf4*, *wtf13*, *wtf19* and *wtf23*. Interestingly, the antidote of one *wtf* driver does not necessarily work against the poison generated by a different *wtf* driver. *cw9* and *cw27*, for example, make incompatible poisons and antidotes such that, when these genes are both heterozygous in a diploid, most of the spores are destroyed (Hu et al., 2017). This observation may underlie the high fraction (77%) of the viable spores produced by *S. pombe*/*S. kambucha* diploids that inherit two (non‐sister) copies of chromosome 3: those spores are more likely to inherit drivers from both strains, thus protecting them from destruction (Zanders et al., 2014). This phenomenon may also explain the failure of the *wtf* gene family to efficiently spread beyond chromosome 3, as this chromosome is the only one for which *S. pombe* tolerates aneuploidy (i.e. one copy of chromosomes 1 and 2, but two copies of chromosome 3; Niwa, Tange, & Kurabayashi, 2006). Aneuploid spores may help mitigate the costs of competing *wtf* drivers on chromosome 3 in a way that could not happen on chromosomes 1 or 2.

Not all *wtf* genes tested, however, appear capable of causing meiotic drive. The *S. kambucha wtf2*, *wtf5*, *wtf6* and *wtf26* genes all appear intact, but they failed to drive when introduced into *S. pombe*. This observation could be because the ability of these genes to drive is suppressed in *S. pombe*, but we favour an alternative hypothesis. These genes all lack an in‐frame start codon near the beginning of exon 2 that could be used to encode a poison. Instead, these genes all appear to encode only one protein that is similar to the antidote of the intact driver genes. This similarity suggests that these and other *wtf* genes that lack the capacity to encode the shorter poison protein make only an antidote (Nuckolls et al., 2017). This antidote could act as a suppressor to other fully intact drivers. Indeed, the landscape of meiotic drivers in the *S. pombe/S. kambucha* hybrids includes drivers as well as suppressors of drive (Nuckolls et al., 2017).

**Figure 2:**
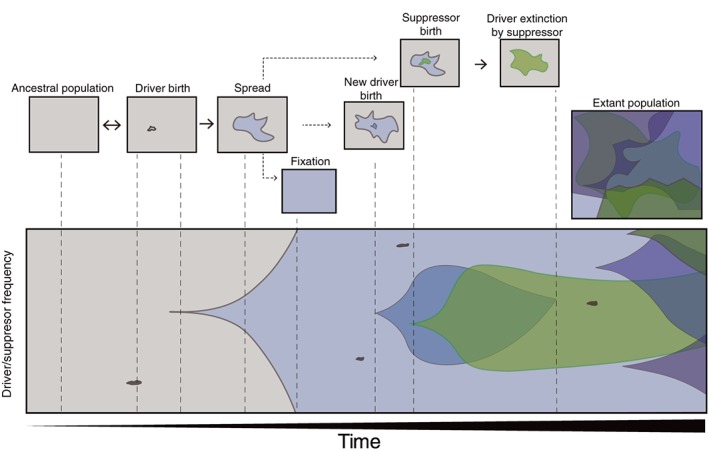
Hypothetical evolutionary history of *wtf* genes in a population over time (left to right). The ancestral population did not carry driving *wtf* genes. The first *wtf* drive gene entered the population by mutation of an existing non‐driving gene, *de novo* gene birth, or by horizontal gene transfer. The *wtf* drive gene can spread in the population and birth new *wtf* drive genes via gene duplication (drivers are shown in shades of blue). Owing to the fitness costs of drivers, suppressors (shown in shades of green) are expected to emerge, perhaps from amongst the *wtf* drive genes themselves, through loss of the poison transcriptional or translational start sites. Fixation of a driver or successful antagonism by a suppressor could each contribute to the pseudogenization of a *wtf* driver. Suppressors lacking targets could also decay. These events are dynamic and ongoing, leading to a mix of functional drivers, suppressors, and pseudogene remnants in the genome [Colour figure can be viewed at http://wileyonlinelibrary.com]

The primordial origin of the *wtf* genes is obscure, but the first *wtf* driver could have been born via mutation of a non‐driving gene. Nuckolls et al. (2017) proposed that such a change preceded the expansion of the family, thus the ancestral function of the expanded family in fission yeast is meiotic drive. The original *wtf* driver gene could have birthed duplicate genes. These duplicate genes could have been maintained owing to their ability to cause drive at a new locus. Eventually, identical *wtf* genes could diverge until their poisons and antidotes no longer neutralized each other, giving rise to distinct, competing selfish drive genes like *cw9* and *cw27* (Hu et al., 2017). Some *wtf* genes (like *S. kambucha wtf2*) could have lost the ability to make a poison but retained the ability to make an antidote protein that could provide protection against intact *wtf* drivers. These *wtf* drive suppressors (and duplicate genes born from them) would have a fitness advantage in a population where drivers are common and should thus be maintained by selection (Crow, 1991). Some *wtf* genes may be maintained at intermediate frequencies whereas others may have spread to fixation in the population. After fixation, a driver loses its selfish advantage and it could decay (poison first) into a pseudogene. Antidote‐only *wtf* suppressor genes could also become pseudogenized if the drivers they antagonize go extinct. A partial hypothetical timeline of *wtf* family evolution is shown in Figure 2.

## 5 WHAT IS THE COST OF SELFISHNESS?

The genetic divergence amongst sequenced isolates of *S. pombe* (>99.5% DNA sequence identity) is similar or lower than what is generally observed in other species such as Saccharomyces cerevisiae (Jeffares et al., 2015). Despite the similarity, it is common to observe reproductive isolation between different *S. pombe* isolates. Hybridization between *S. pombe* strains often yields <50% viable offspring (Avelar, Perfeito, Gordo, & Ferreira, 2013; Hu et al., 2015; Jeffares et al., 2017; Zanders et al., 2014). This is in sharp contrast with *Saccharomyces sensu stricto* yeast where crosses for genotypes with >99% similarity generally have high fertility (Hou, Friedrich, de Montigny, & Schacherer, 2014). This suggests that there are forces driving extremely rapid evolution leading to reproductive isolation in fission yeasts (within ~2300 years; Jeffares et al., 2015).

The *wtf* meiotic drivers are a major cause of infertility in laboratory *S. pombe*/*S. kambucha* and laboratory *S. pombe*/CBS5557 hybrids (Hu et al., 2017; Nuckolls et al., 2017). It is also plausible that *wtf* genes are a key underlying cause of hybrid infertility between other *S. pombe* isolates. Given the large number of *wtf* loci and their rate of change, it seems likely that one or more distinct driving *wtf* genes will be heterozygous in any given hybrid. These heterozygous *wtf* genes would each have the potential to kill up to half of the progeny.

The other verified cause of *S. pombe* hybrid infertility is chromosome rearrangements (Avelar et al., 2013; Hu et al., 2015; Jeffares et al., 2017; Zanders et al., 2014). Karyotype changes are common in *S. pombe*: changes dramatic enough to be detected on pulse‐field gels were present in ~20% of natural isolates (Brown et al., 2011; Jeffares et al., 2017). The high level of karyotype diversity in the *S. pombe* population is remarkable because of the high reproductive costs of chromosome rearrangements when heterozygous. Why have these rearrangements been tolerated by selection?

There are several, non‐mutually exclusive explanations for this paradox. One is that rearrangements can give rise to beneficial phenotypes and be maintained by selection (Avelar et al., 2013; Jeffares et al., 2017). Another explanation is that *S. pombe* could infrequently outcross, minimizing the fertility costs of rearrangements. Population genetic analyses do provide evidence of outcrossing, but the true frequency of outcrossing is difficult to gauge because meiotic drive can minimize evidence of outcrossing (Farlow et al., 2015; Jeffares et al., 2015). *S. pombe/S. kambucha* hybrids, for example, transmit predominantly *S. kambucha* alleles on all three chromosomes (Zanders et al., 2014). The notable evolutionary success of the *wtf* gene family also supports outcrossing. Meiotic drivers rely on heterozygosity, and thus frequent outcrossing. Without it, drivers lose the opportunity to act and should go extinct. The expansion and maintenance of the *wtf* gene family therefore argues in favour of frequent outcrossing. We propose that chromosome rearrangements are sometimes maintained or even spread in the population because of their genetic linkage to meiotic drive alleles (Zanders et al., 2014).

It is interesting to speculate how *S. pombe* will bear the burden of a genome full of *wtf* meiotic drive parasites over evolutionary time (Figure 2). The high likelihood of spores being destroyed by drive or lacking essential genes owing to rapid karyotype evolution raises the question of why *S. pombe* even bothers outcrossing? These factors must be exerting significant evolutionary pressure to shape sexual reproduction. Perhaps variants that eschew traditional outcrossing in favour of other stress response or parasexual pathways could be favoured by selection? Perhaps a universal suppressor of *wtf* drivers will arise and drive these selfish parasites extinct? Perhaps *S. pombe* isolates with *wtf* genes will be unable to overcome their parasite burden and the species will go extinct?

## 6 FUTURE PERSPECTIVES

Innumerable areas of scientific inquiry have been founded on discoveries made in yeasts. Yet in the meiotic drive field, the yeast drive genes were not discovered until 60 years after meiotic drive was formally defined. Despite this lag phase, the drive field is still quite young in terms of molecular understanding and yeast has much to contribute. The *wtf* genes are not widely found in eukaryotes, but killer meiotic drive loci are (Burt & Trivers, 2006; Lindholm et al., 2016). As meiotic drivers in different organisms are not orthologous, they will not necessarily use the same mechanisms as *wtf* genes. It is possible, however, that through convergent evolution some drivers will use similar molecular mechanisms or target similar vulnerable aspects of gametogenesis. For instance, the *het‐s* drive system causes spore death via membrane disruption, analogous to the proposed mechanism of *wtf* action (Seuring et al., 2012; Figure 1). In addition, the yeast system will facilitate high‐throughput empirical analyses of meiotic driver and suppressor evolution that are largely intractable in non‐microbial systems. Despite arriving late to the party, yeasts may yet guide discovery and analyses of meiotic drive systems in more complex eukaryotes, including humans.

## CONFLICTS OF INTEREST

S.E.Z. is an inventor on a patent application based on *wtf* drivers, serial 62/491,107. The authors declare that there are no other conflicts of interest.
